# A case report on death from acute bacterial cholangitis accompanied by von Meyenburg complexes: Use of 16S rRNA gene sequencing to identify pathogenic microbes from postmortem formalin-fixed, paraffin-embedded tissue

**DOI:** 10.1097/MD.0000000000025526

**Published:** 2021-04-16

**Authors:** Noriko Watanabe, Shin-ichiro Ohno, Moe Sakuma, Mayo Kuriwaki, Mai Miura, Masahiko Kuroda

**Affiliations:** Department of Molecular Pathology, Tokyo Medical University, Shinjuku-ku, Tokyo 160–8402, Japan.

**Keywords:** 16S ribosomal RNA, acute cholangitis, autopsy, formalin-fixed paraffin-embedded, von Meyenburg complexes

## Abstract

**Rationale:**

In some cases, autopsy is the first opportunity to find a previously unrecognized critical infection. Pathogens are identified by various methods, such as microscopic examination, special stains, culture tests, and immunohistochemistry. Here, we report a case of 16S ribosomal RNA (rRNA) gene sequencing using a postmortem formalin-fixed, paraffin-embedded (FFPE) tissue, which was useful for identifying pathogenic microbes.

**Patient concerns:**

Autopsy was performed on an 87-year-old man who had chronic renal failure and had developed sepsis from a central venous catheter infection 10 days before his death. Prior to these events, von Meyenburg complexes (VMCs) were also found during regular checkups.

**Diagnosis:**

Postmortem microscopic examination revealed acute purulent cholangitis with numerous microabscesses, accompanied by VMCs. Gram-negative rods were observed in some microabscesses, which were considered causative pathogens.

**Interventions:**

16S rRNA gene sequencing using postmortem FFPE tissue

**Outcomes:**

*Pseudomonas aeruginosa* was identified, different from the one detected in the central venous catheter culture while alive.

**Lessons:**

16S rRNA gene sequencing is a useful tool for identifying pathogenic microbes in postmortem FFPE tissues. This technique may be useful for amplicon sizes of approximately 100 bp or less.

## 1 Introduction

Deaths caused by infectious diseases accounted for 3 out of the top 10 causes of death worldwide in 2019, as reported by the World Health Organization.^[1]^ Naturally, we often encounter autopsies presenting with infection as the cause of death. Pathogens can be identified via morphological approaches and culture tests with pathogen-specific markers, depending on the case. The 16S ribosomal RNA (rRNA) gene sequencing with universal bacterial primers is a useful tool to identify bacterial pathogens without targeting a specific pathogen. It can be performed using formalin-fixed, paraffin-embedded (FFPE) tissues, which have been in use since the 1990s.^[2–10]^ This technique is especially effective for cases of culture-negative bacteria that often occur in infective endocarditis cases or culture-difficult bacteria. However, such cases are mostly limited to biopsy or surgically resected samples that are collected under sterilized conditions and freshly processed, suggesting that 16S rRNA gene sequencing using FFPE tissues obtained by autopsy is challenging. In autopsy cases, elongated formalin fixation is known to result in DNA degradation.^[11,12]^ In addition, there is a risk of bacterial contamination, possibly from the postmortem proliferation of resident flora in patients or through its introduction during the autopsy and FFPE processing.

Here, we report a case in which microscopic examination of autopsied specimens revealed acute bacterial cholangitis with numerous microabscesses accompanied by von Meyenburg complexes (VMCs). By performing 16S rRNA gene sequencing on a sample from the same postmortem paraffin block as Gram-negative rods were observed in, *Pseudomonas aeruginosa* (*P. aeruginosa*) was listed as the most potential pathogen. This was confirmed by a *P. aeruginosa* DNA amplification followed by sequencing, and *P. aeruginosa*-specific immunohistochemical staining.

## 2 Case presentation

**Figure 1:**
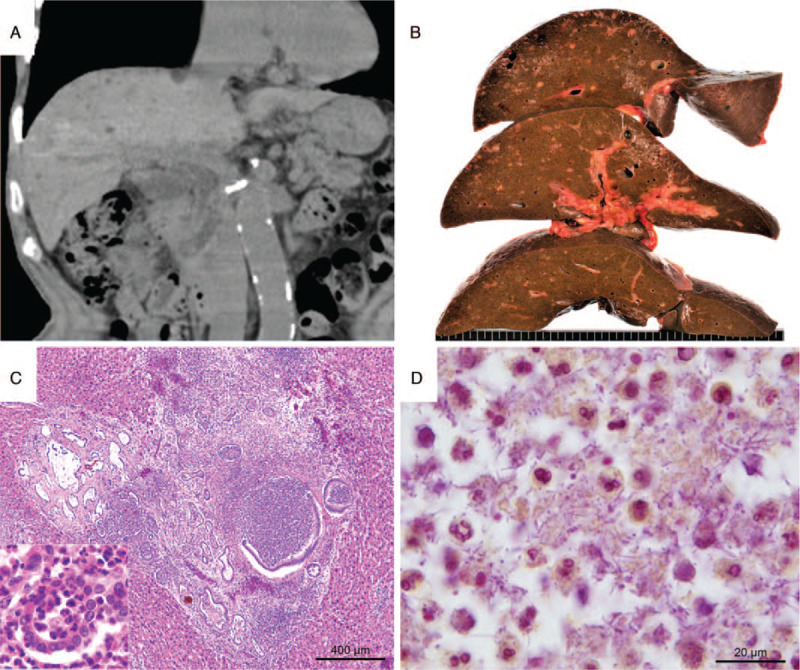
Hepatic findings of the patient. (A) Computed tomography (CT) taken prior to admission presented multiple small hypodense lesions scattered in the liver, especially in the subcapsular area of right lobe. (B) During autopsy, a number of small nodules and cysts were found scattered in the liver, predominantly in the subcapsular area of right lobe, which seemed to be consistent with the CT findings. (C) Microscopic findings revealed an enlarged portal area with several irregularly-dilated bile ducts containing aggregated neutrophils (inset), partially infiltrated to hepatic parenchyma (HE stain). (D) Some microabscesses contained gram-negative rods (gram stain using the Brown and Hopps method).

The patient was an 87-year-old man who had presented with hypercalcemia at a health check 3 years before, resulting in parathyroid adenoma found by 99mTc-MIBI scintigraphy. He was prescribed bisphosphonate and regularly followed. During follow-up, he was also diagnosed with VMCs based on findings of multiple small hypodense lesions in the liver as observed on plain computed tomography (Fig. 1A). Two months prior to his death, he was hospitalized for a hypercalcemia crisis presenting dehydration, hypercalcemia (serum calcium, 15.9 mg/dL), and renal dysfunction (serum creatinine, 2.7 mg/dL). Hypercalcemia was improved by intravenous rehydration and synthetic calcitonin administration, although renal dysfunction remained. During hospitalization, he repeatedly suffered from aspiration pneumonitis and pseudomembranous colitis, each of which was improved by antibiotic administration. Ten days prior to his death, he developed septic fever. *Ochrobactrum anthropic* was detected in both blood and central venous catheter cultures. The central venous catheter was removed and meropenem was administered; however, his fever continued and he died. An autopsy was performed 18 hours after his death. Grossly, a large number of small lesions consisting of nodules and cysts were observed in the liver (Fig. 1B). They were considered VMCs; therefore, culture tests were not performed. Microscopically, the lesions presented as acute purulent cholangitis with numerous microabscesses accompanied with VMCs (Fig. 1C). In some microabscesses, gram-negative rods were observed (Fig. 1D), especially in the hepatic portal area, and in the common bile duct, which indicated that pathogenic bacteria had ascended from the bowel. Initially, we suspected the bacteria of *Ochrobactrum anthropic*, which were detected in both blood and central venous catheter cultures 10 days before death. However, if that was the case, the bacteria should have infected via blood vessels, which was inconsistent with the microscopic findings. Therefore, we conducted 16S rRNA gene sequencing using the postmortem FFPE tissues.

**Figure 2:**
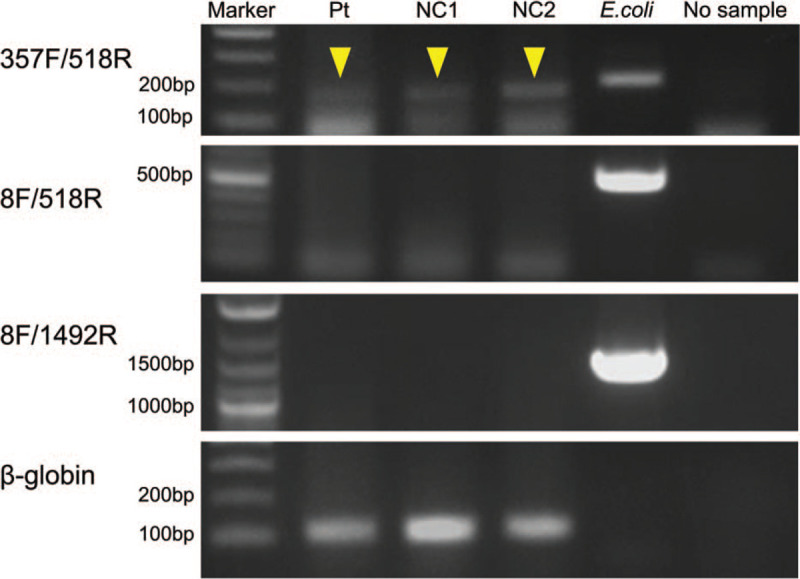
PCR results. Amplified products using the 357F/518R primer pair of the 16S rRNA gene appeared as a faint band in the patient (Pt) and as faint bands with slightly larger size in the 2 negative control cases (NC1 and NC2) (yellow arrows, top panel). Both 8F/518R and 8F/1492R primer pairs of the 16S rRNA gene resulted in no bands at all, except for the positive control DNA extracted from cultured *Escherichia coli* (middle panels). Human β-globin primers for a 110-bp amplicon resulted in positive bands in all postmortem cases (bottom panel).

Detailed methodology is provided in the supplemental material. We selected FFPE liver tissue showing acute cholangitis with abundant gram-negative rods in microabscesses. For the negative control, 2 noninfectious FFPE liver tissues, obtained from 2 different autopsy cases, were used. All samples underwent FFPE processing after approximately 1 month of fixation with 20% buffered formalin. Three sets of primer pairs for the 16S rRNA gene with different amplicon sizes were used: 357F/518R, 8F/518R, and 8F/1492R. To assess the human DNA preservation status in the FFPE tissues, a human β-globin primer pair was used. Amplicon from PCR with 357F/518R primer pair appeared as a faint band in this case and as faint bands with a slightly larger size in the 2 negative control cases (Fig. 2, top panel). DNA sequencing of this case revealed the *Pseudomonas* genus was the most likely causative bacteria, as determined by the NCBI BLAST database (http://www.ncbi.nlm.nih.gov/BLAST/). Among them, *P. aeruginosa* was suspected to be the prime pathogen, with 85% similarity to the amplified 100-bp band. Meanwhile, sequences from the negative control cases showed a very weak matching to the various genera, and was considered to be nonspecifically amplified DNA. PCR products using both primer pairs, 8F/518R and 8F/1492R, showed no bands (Fig. 2, middle panels), which suggested DNA fragmentation in postmortem FFPE samples, and also implied no contamination by fresh bacteria during the FFPE processing. PCR products using human β-globin primers for a 110-bp PCR product showed positive bands for all postmortem cases (Fig. 2, bottom panel).

**Figure 3:**
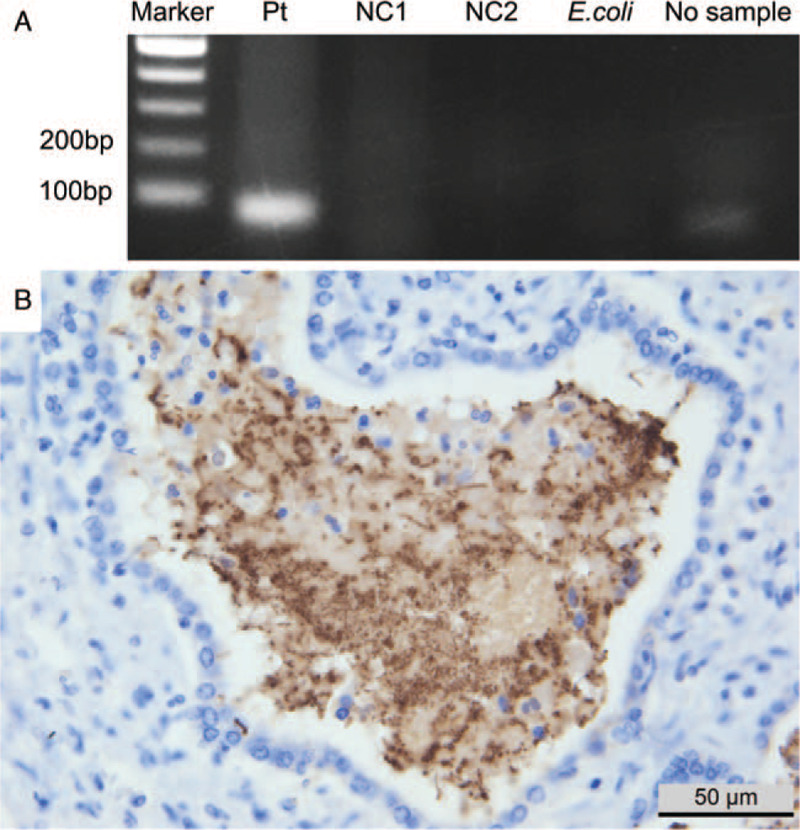
Confirmation of *Pseudomonas aeruginos*a. (A) PCR products using primers for the *oprL* gene with 72-bp amplicon size showed positive band for the patient (Pt) and no bands for 2 negative control cases (NC1 and NC2). (B) Immunohistochemical staining for anti-*Pseudomonas* antibody was positive for rod-shaped bacteria in the microabscess.

To further confirm *P. aeruginosa*, we performed *P. aeruginosa*-specific DNA amplification followed by sequencing and immunohistochemical staining. PCR products using the primer pair for a 72-bp amplicon of the *oprL* gene showed a clear band for only this case (Fig. 3A). The resultant sequence revealed 100% concordance with *P. aeruginosa*. Immunohistochemistry for anti-Pseudomonas antibody (ab68538, rabbit polyclonal, 1:1000 dilution; Abcam, Cambridge, UK) showed distinctly positive results for rod-shaped bacteria in the microabscess (Fig. 3B).

## 3 Discussion

von Meyenburg complexes (VMCs), also known as biliary hamartoma, characterized by microscopic findings of small bile duct aggregates surrounded by fibrous stroma, are reported to be found in 5.6% of adult autopsy cases.^[13]^ As imaging technology improves, more cases are diagnosed without histological confirmation. Most cases are asymptomatic and rarely present hepatobiliary infectious complications that could be life-threatening.^[14]^ In our case, acute purulent cholangitis accompanied by VMCs caused by *P. aeruginosa*, which presented as an infection through the intestinal tract, was considered as the direct cause of death, based on the weekended immune state of the patient owing to chronic renal dysfunction, malnutrition, aging, and repetitive infectious episodes during hospitalization.

Occasionally, an autopsy is the first opportunity to recognize a critical infection. In addition to conventional methods including microscopic examination with special stains, 16S rRNA gene sequencing with universal bacterial primers is useful in identifying pathogenic microorganisms, which can be performed using the same paraffin block as the one in which pathogenic microbes were observed in. However, few cases have reported successful execution from autopsy FFPE specimens. One case identified oral *Streptococcus* species in infective endocarditis,^[5]^ and another, which is included in the case series study, detected *Staphylococcus epidermidis* from liver lesions of abscess *vs.* cancer.^[9]^ In general, FFPE tissues obtained by autopsy show severe DNA fragmentation, which hampers PCR analysis. Zhi et al reported only a 23.1% positive PCR rate using a 110-bp amplicon β-globin primer set in lung FFPE tissues obtained from 65 autopsy cases, which was not correlated with the postmortem interval and storage term of paraffin blocks.^[15]^ They estimated that long-term formalin fixation may promote DNA fragmentation. Actually, Vitošević et al reported that DNA fragmentation in autopsy FFPE tissues proceeded according to the period of formalin fixation.^[11]^ Bonin et al mentioned that the average fragment length of DNA is 300 to 400 bases in biopsy FFPE tissues, but much shorter in postmortem FFPE tissues, most often less than 100 bases.^[12]^ This was also the case with our study, as our PCR analyses using primer pairs for a >200-bp product showed negative results, whereas for ≤110-bp producing primer pairs, the results were positive and stable.

The lessons learnt from this case study are as follows:

1. universal primers for the 16S rRNA gene would be better for amplicon sizes of approximately ≤100 bp;
2. to decrease the risk of bacterial contamination, the microtome and tweezers should be sterilized, blade should be changed to a new one between blocks, and reagents should be aseptic;
3. postmortem FFPE tissue without infection should be used as a negative control and should be processed simultaneously until sequencing analysis; and
4. a confirmation step using markers specific to the identified bacteria may be necessary for a precise evaluation.

Point 1 should be optimized using a variety of primers, because the degree of DNA fragmentation varies depending on the case, as formalin fixation protocols differ between institutes. Points 2 to 4 are important to avoid confusion in the interpretation of 16S-amplicon sequencing results, and to acquire convincing results.

## Acknowledgments

We thank Dr Kanekura, Assistant Professor of Department of Molecular Pathology, Tokyo Medical University for editing a draft of this manuscript.

## Author contributions

**Conceptualization:** Noriko Watanabe.

**Investigation:** Noriko Watanabe, Moe Sakuma, Mayo Kuriwaki, Mai Miura.

**Methodology:** Shin-ichiro Ohno.

**Supervision:** Masahiko Kuroda.

**Writing – original draft:** Noriko Watanabe.

**Writing – review & editing:** Masahiko Kuroda.

## Supplementary Material

Supplemental Digital Content
