# Supplementary material for: A case report on death from acute bacterial cholangitis accompanied by von Meyenburg complexes: Use of 16S rRNA gene sequencing to identify pathogenic microbes from postmortem formalin-fixed, paraffin-embedded tissue
Source: Medicine (Baltimore). 2021 Apr 16;100(15):e25526. doi: 10.1097/MD.0000000000025526 (PMC8051965; doi:10.1097/MD.0000000000025526)
Supplement: Supplemental Digital Content [file medi-100-e25526-s001.docx]

Supplementary material

A case report on death from acute bacterial cholangitis accompanied by von Meyenburg complex: use of 16S rRNA gene sequencing to identify pathogenic microbes from postmortem formalin-fixed, paraffin-embedded tissue

Noriko Watanabe

Methodology

For 16S rRNA gene sequencing, four 5-μm thick sections of approximately 400 mm^2^ surface area were cut into scrolls using a microtome and tweezers, which were sterilized with alcohol, and the blade was changed for each sample. DNA was extracted using the QIAamp DNA FFPE Tissue Kit with Deparaffinization Solution (Qiagen, Hilden, Germany) following the manufacturer’s instructions, with extended incubation with proteinase K overnight. The bacterial 16S rRNA-encoding gene was amplified by PCR using Takara Ex Taq (Takara Bio Inc., Kusatsu, Japan) DNA polymerase following the manufacturer’s protocol with thermal cycling conditions of initial denaturation at 98 **°**C for 1 min followed by 35 cycles of denaturation at 98 **°**C for 10 s, annealing at 55 **°**C for 30 s, and extension at 72 **°**C for 1 min, followed by a final extension at 72 **°**C for 5 min. All reagents were sterilized with a membrane filter and prepared in a laminar flow cabinet to prevent bacterial contamination. Three sets of universal primer pairs for the16S rRNA gene with different amplicon sizes were used: 357F/518R, 8F/518R, and 8F/1492R. For assessment of human DNA preservation status in FFPE tissues, a human β-globin primer pair with an amplicon size of 110-bp was used. For *P. aeruginosa*-specific DNA amplification, a primer pair for the *oprL* gene with amplicon size of 72-bp was used. The primer sequences are shown in Table at the end of this document. The amplified products were visualized on 1% or 2% agarose gels by electrophoresis with ethidium bromide staining. The amplified bands were excised, purified with the QIAquick Gel Extraction Kit (Qiagen), and sequenced using commercial Sanger sequencing services (https://www.genewiz.com).

For the immunohistochemical staining, 4-μm-thick FFPE sections were immersed in hydrogen peroxidase (0.3% in methanol) for 15 min to quench endogenous peroxidase activity, and antigen retrieval was performed by autoclaving at 121 **°**C for 10 min in citrate buffer at pH 6.0. After cooling, the sections were incubated with rabbit polyclonal anti-Pseudomonas antibody (ab68538, 1:1000 dilution; Abcam, Cambridge, UK) for 60 min at room temperature, followed by incubation with a horseradish peroxidase-labeled anti-rabbit secondary antibody for 30 min at room temperature. Sections were stained with 3,3-diaminobenzidine for visualization, and then counterstained with hematoxylin.

Table List of primer sequence

| Target gene | Sequence (5’-3’) | Reference |
| --- | --- | --- |
| 16S rRNA |  |  |
| 8F | AGAGTTTGATCCTGGCTCAG | http://www.bexnet.co.jp |
| 357F | CTCCTACGGGAGGCAGCAG | [1] |
| 518R | GTATTACCGCGGCTGCTGG | [2] |
| 1492R | GGCTACCTTGTTACGACTT | http://www.bexnet.co.jp |
| β-globin |  |  |
| F | ACACAACTGTGTTCACTAGC | [3] |
| R | CAACTTCATCCACGTTCACC | [3] |
| *oprL* |  |  |
| F | AGCCTTCCTGGTCCCCTTAC | [4] |
| R | CCTAATGAACCCCAGTGTATAAGTTTG | [4] |

[1] Turner S, Pryer KM, Miao VP, et al. Investigating deep phylogenetic relationships among cyanobacteria and plastids by small subunit rRNA sequence analysis. J Eukaryot Microbiol 1999; 46: 327–38.

[2] Lane DJ. 16S/23S rRNA sequencing. In: Stackebrandt E, Goodfellow M, eds. Nucleic acid techniques in bacterial systematics. New York: John Wiley & Sons, 1991; 115-75.

[3] Saiki RK, Stoffel S, Scharf SJ, et al. Primer-directed enzymatic amplification of DNA with a thermostable DNA polymerase. Science 1988; 239: 487–91.

[4] Fukumoto H, Sato Y, Hasegawa H, et al. Development of a new real-time PCR system for simultaneous detection of bacteria and fungi in pathological samples. Int J Clin Exp Pathol 2015; 8: 15479–88.
